# RNA interference therapy for targeting ANGPTL3 and atherogenic lipoproteins: Findings and implications of a recent phase I study

**DOI:** 10.1002/ctm2.1484

**Published:** 2023-11-27

**Authors:** Gerald F. Watts, Dick C. Chan

**Affiliations:** ^1^ Medical School University of Western Australia Perth Western Australia Australia; ^2^ Department of Cardiology and Internal Medicine Cardiometabolic Service Royal Perth Hospital Perth Western Australia Australia

COMMENTARY:

ANGPTL3 (angiopoietin‐like protein 3) is a hepatokine exclusively secreted by the liver and a key regulator of plasma lipid and lipoprotein metabolism through the inhibition of lipoprotein lipase (LPL) and endothelial lipase (EL).^1^ Mendelian randomization studies show that carriers of loss‐of‐function mutations in *ANGPTL3* have on average a 35% reduction in the lifetime risk of atherosclerotic cardiovascular disease (ASCVD) events.^2^ Genetic deficiency in ANGPTL3 does not have adverse clinical sequelae. Recent development of RNA interference (siRNA) therapy targeting ANGPTL3 offers a new approach for lowering plasma triglyceride and low‐density lipoprotein‐cholesterol (LDL‐C) concentrations.^3^, ^4^ ARO‐ANG3 is the most advanced tri‐antennary *N*‐acetylgalactosamine (GalNAc)‐conjugated siRNA targeted at the liver, where it specifically inhibits and degrades ANGPTL3 mRNA in the RNA‐induced silencing complex within the cytoplasm.

## 1 NEW PHASE I CLINICAL TRIAL WITH ARO‐ANG3 USING RNA INTERFERENCE

**FIGURE 1:**
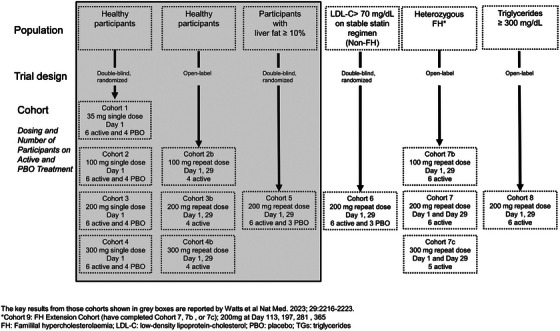
Basket trial design of phase 1 study of ANGPTL3 inhibition by ARO‐ANG3 (AROANG1001). The cohorts included in the recent publication by Watts et al.^5^ are highlighted.

AROANG1001 is the first‐in‐human, phase 1 basket trial design that evaluated the safety, tolerability, pharmacokinetic and pharmacodynamic effects of ARO‐ANG3 in adult healthy and dyslipidaemic subjects (Figure 1).^5^ An analysis of the effect of ARO‐ANG3 in 52 healthy participants (Cohorts 1–4) and nine participants with hepatic steatosis, defined as baseline magnetic resonance–proton density fraction (MRI‐PDFF) ≥10% (Cohort 5), was reported recently.^5^

**FIGURE 2:**
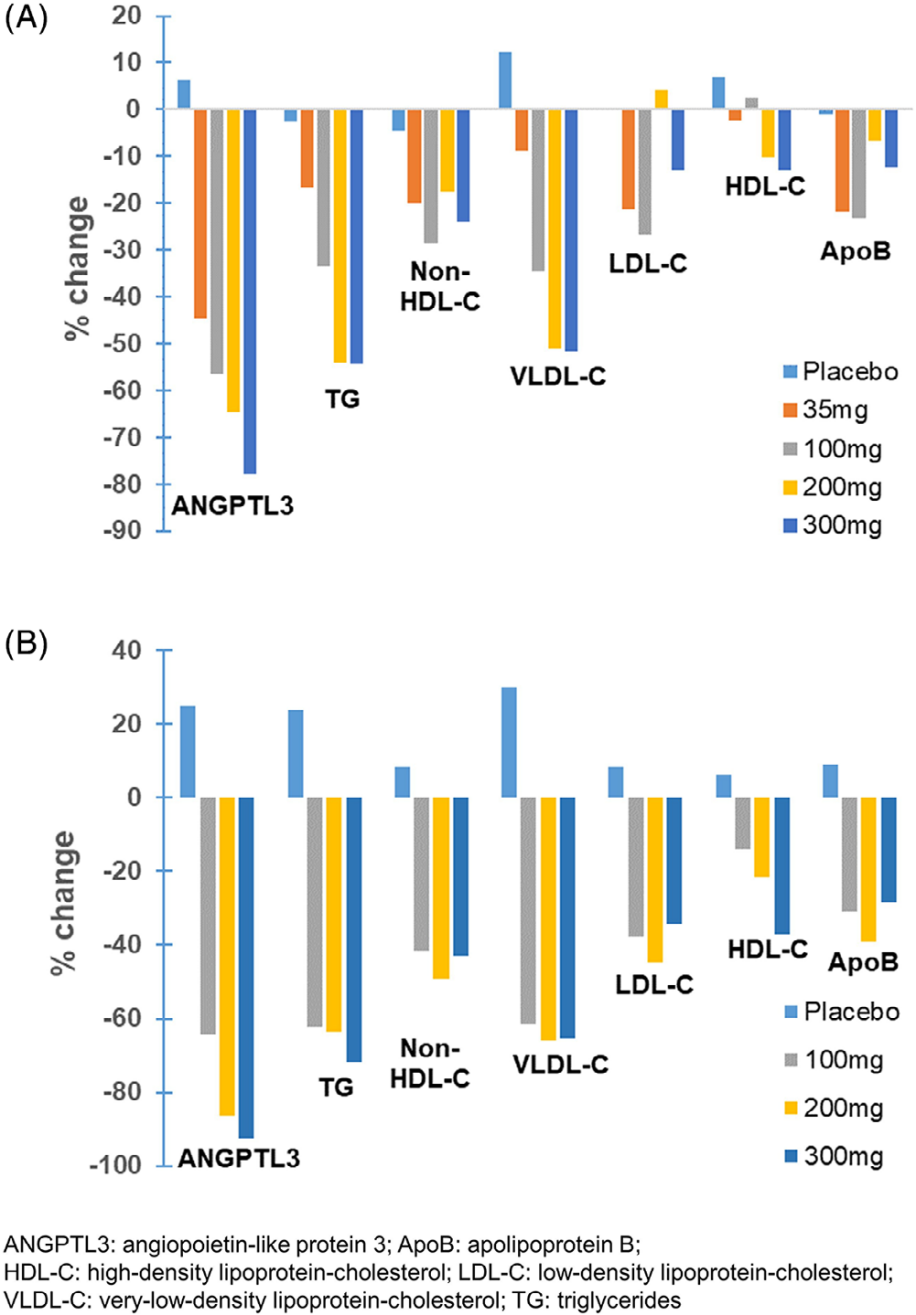
Percentage change relative to baseline in serum ANGPTL3 and key lipid and lipoprotein parameters with (A) single‐ascending and (B) repeat‐ascending doses of ARO‐ANG3 in healthy participants.^5^

Briefly, a single dose of ARO‐ANG3 reduced serum ANGPTL3 (mean −57% to −78%), triglyceride (median −34% to −54%), non‐high‐density lipoprotein cholesterol (non‐HDL‐C) (mean −24% to −29%), very‐low‐density lipoprotein‐cholesterol (VLDL‐C) (mean −35% to −52%) and LDL‐C (mean +4.1% to −27%) concentrations with the three highest dose (100, 200 and 300 mg) 85 days after dosing (Figure 2A). Repeat dosing of ARO‐ANG3 (Day 1 and Day 29) achieved a substantial and sustained decrease in serum ANGPTL3 (−64% to −93%), triglyceride (−62% to −72%), non‐HDL‐cholesterol (−41% to −49%), VLDL‐C (−62% to −66%) and LDL‐C (−34% to −45%) concentrations from baseline to Day 113 (Figure 2B). Single and multiple dose‐escalating effects for ANGPTL3 and key lipid parameters in Cohorts 1−4 are summarized as relative changes from baseline in Figure 2A,B, respectively. There were no deaths, life‐threatening treatment‐emergent adverse events (TEAEs), or TEAEs leading to drug discontinuation or premature withdrawal of any participant from the trial. In the same report, a repeat dose of ARO‐ANG3 (Day 1 and Day 29) also achieved a substantial and sustained decrease in serum ANGPTL3 (−85%), triglyceride (−47%), non‐HDL‐cholesterol (−37%), VLDL‐C (−43.5%) and LDL‐C (−35%) concentrations, with the 200 mg dose in participants with baseline hepatic steatosis (Cohort 5), with no meaningful adverse changes in liver fat observed with ARO‐ANG3 treatment. The most frequently reported TEAEs were mild injection site reactions, which are common to all subcutaneous injections. The results from this early‐stage clinical trial indicate that siRNA therapy targeting ANGPTL3 mRNA is safe, well tolerated and can effectively lower serum concentrations of atherogenic lipoproteins, specifically triglyceride‐rich lipoproteins (TRLs) and LDLs. These encouraging results support the development of this novel therapy for testing in phase 2 and 3 clinical trials.

## 2 THERAPEUTIC PERSPECTIVE AND FUTURE RESEARCH

Elevated plasma triglyceride and LDL‐C concentrations are both causally related to ASCVD risk.^6^ ANGPTL3 is a genetically validated target for lowering both TRLs and LDLs. siRNA‐based therapies targeting ANGPTL3 may accordingly reduce residual ASCVD risk in dyslipidaemic patients receiving best standard‐of‐care treatment.^3^, ^4^ An ongoing phase 2 trial in adults with mixed dyslipidaemia (NCT04832971) is currently underway to evaluate the efficacy and safety of ARO‐ANG3.^7^

Given that the mechanism of action of ANGPTL3 inhibition with ARO‐ANG3 appears to be independent of the LDL receptor, this agent may be a significant advance for managing patients with severe hypercholesterolaemia, particularly in those with homozygous FH (HoFH) who have minimal or no residual LDL receptor function.^4^ Achieving acceptable LDL‐C goals in HoFH patients without the need for lipoprotein apheresis remains a major challenge in clinical practice. The sustained efficacy and safety of evinacumab, an anti‐ANGPTL3 monoclonal antibody, have been shown in the ELIPSE HoFH trial, with a mean LDL‐C reduction of 49% following 24 weeks of therapy.^8^ Accordingly, there is an ongoing phase 2 dose‐finding study in HoFH patients with ARO‐ANG3 (NCT05217667). Whether similar efficacy can be achieved with this agent remains to be confirmed.^9^

ANGPTL3 inhibition with siRNA‐based therapy is not only an effective therapeutic option for severe FH and mixed dyslipidaemia, but may also be useful for mitigating hepatic steatosis associated with the insulin resistance syndrome.^3^ Increase in the clearance of chylomicrons and other triglyceride‐rich lipoproteins (TRLs) with inhibition of ANGPTL3 may also provide effective treatment for severe hypertriglyceridaemia and the prevention of acute pancreatitis.

## 3 CONCLUSION: RESEARCH QUESTIONS

Despite optimal lifestyle and statin therapies, a substantial proportion of patients, mostly those with FH, do not reach guideline‐recommended LDL‐C goals. There is also a substantial residual risk of ASCVD attributable to other atherogenic lipoproteins, such as TRLs.^6^ Hence, there is a major need to close this gap in care using treatments that lower LDL‐C and TRLs via novel mechanisms of action. The robust and sustained effect in lowering serum TRLs and LDL with gene silencing targeted at ANGPTL3 meets this need. ARO‐ANG3 may also close a major treatment gap in lack of adherence to treatment related to its pharmacokinetic effect that achieves a prolonged duration of action. However, further research is required to understand the exact mode of action of ARO‐ANG3 on the metabolism of atherogenic lipoproteins.^3^ The impact of hepatic inhibition of ANGPTL3 on hepatic VLDL secretion and insulin‐mediated VLDL assembly/secretion also warrants further investigation, as well as the effect on HDL metabolism. Although apoC‐III inhibition appears to have greater efficacy in lowering elevated plasma triglycerides, ANGPTL3 inhibition may have the advantage of greater lowering of plasma LDL‐C and apoB concentrations. ARO‐ANG3 may have a special role in lowering elevated LDL‐C in FH refractory to standard drug therapies, including proprotein convertase subtilisin/kexin type 9 (PCSK9) inhibitors. This needs verification in ongoing clinical trials. The molecular mechanisms whereby ANGPTL3 inhibition influences both hepatic and peripheral insulin resistance and the overall effects on glucose metabolism and hepatic steatosis require further investigation. It is noteworthy that by contrast to an antisense (single‐stranded DNA) oligonucleotide approach (vupanorsen),^10^ no increase in hepatic steatosis has been reported with ARO‐ANG3. The long‐term safety and cost‐effectiveness of treatment with ARO‐ANG3 in addressing residual risk of ASCVD compared with current best standard of care in patients with hypercholesterolaemia and mixed hyperlipidaemia remains to be demonstrated in future studies.

## CONFLICT OF INTEREST STATEMENT

Gerald F. Watts has received honoraria for advisory boards and research grants from Amgen, Arrowhead, Esperion, AstraZenca, Kowa, Novartis, Pfizer, Sanofi and Regeneron. Dick C. Chan declares no conflict of interest.
